# Triple Negative Breast Cancer and Breast Epithelial Cells Differentially Reprogram Glucose and Lipid Metabolism upon Treatment with Triterpenic Acids

**DOI:** 10.3390/biom10081163

**Published:** 2020-08-08

**Authors:** Ângela R. Guerra, Ana F. Paulino, Maria M. Castro, Helena Oliveira, Maria F. Duarte, Iola F. Duarte

**Affiliations:** 1 Department of Chemistry, CICECO-Aveiro Institute of Materials, University of Aveiro, 3810-193 Aveiro, Portugal; angela.guerra@ua.pt; 2 Centro de Biotecnologia Agrícola e Agro-Alimentar do Alentejo (CEBAL)/Instituto Politécnico de Beja (IPBeja), Apartado 6158, 7801-908 Beja, Portugal; ana.paulino@cebal.pt (A.F.P.); c.mariamiguel@gmail.com (M.M.C.); 3 Department of Biology & CESAM, University of Aveiro, 3810-193 Aveiro, Portugal; holiveira@ua.pt; 4 MED—Mediterranean Institute for Agriculture, Environment and Development, CEBAL, 7801-908 Beja, Portugal

**Keywords:** betulinic acid, ursolic acid, triple negative breast cancer, cell metabolism, NMR metabolomics

## Abstract

Plant-derived pentacyclic triterpenic acids (TAs) have gained increasing attention due to their multiple biological activities. Betulinic acid (BA) and ursolic acid (UA) modulate diverse pathways in carcinogenesis, offering increased changes of success in refractory cancers, such as triple negative breast cancer (TNBC). The present work aimed to assess the metabolic effects of BA and UA in MDA-MB-231 breast cancer cells (TNBC model), as well as in MCF-10A non-cancer breast epithelial cells, with a view to unveiling the involvement of metabolic reprogramming in cellular responses to these TAs. Cell viability and cell cycle analyses were followed by assessment of changes in the cells exo- and endometabolome through ^1^H NMR analysis of cell culture medium supernatants, aqueous and organic cell extracts. In MDA-MB-231 cells, BA was suggested to induce a transient upregulation of glucose consumption and glycolytic conversion, tricarboxylic acid (TCA) cycle intensification, and hydrolysis of neutral lipids, while UA effects were much less pronounced. In MCF-10A cells, boosting of glucose metabolism by the two TAs was accompanied by diversion of glycolytic intermediates to the hexosamine biosynthetic pathway (HBP) and the synthesis of neutral lipids, possibly stored in detoxifying lipid droplets. Additionally, breast epithelial cells intensified pyruvate consumption and TCA cycle activity, possibly to compensate for oxidative impairment of pyruvate glycolytic production. This study provided novel insights into the metabolic effects of BA and UA in cancer and non-cancer breast cells, thus improving current understanding of the action of these compounds at the molecular level.

## 1. Introduction

Triple negative breast cancer (TNBC) comprises invasive breast tumors which lack the expression of estrogen receptor (ER), progesterone receptor (PR) and human epidermal growth factor receptor type 2 (HER2) overexpression/HER2 gene amplification. TNBC represents 10–20% of all breast cancer cases and is often diagnosed in women younger than 60 years of age [1]. It is a highly heterogeneous disease, which presents higher relapse rates and poorer clinical outcomes than other breast cancer subtypes [2]. At present, there are no targeted therapies available for TNBC, and chemotherapy remains the only therapeutic option for affected individuals [3,4,5], having failed to improve patient survival [6]. Hence, the search for new drugs or adjuvant treatment options remains a pressing challenge in the management of TNBC.

**Figure 1:**
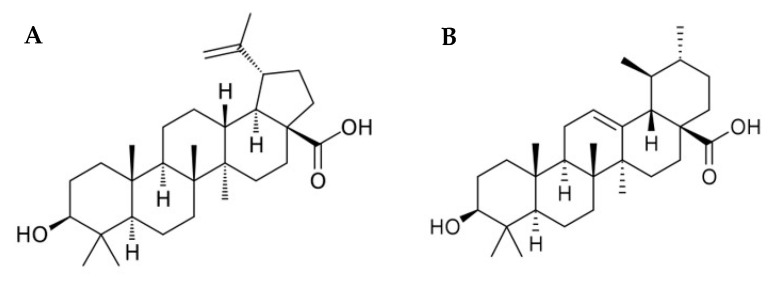
Structural formula of (**A**) betulinic acid and (**B**) ursolic acid.

Natural products have historically driven the discovery of new drugs by the pharmaceutical industry, mainly due to their high structural diversity and remarkable biological activities [7,8]. Triterpenoids represent a numerous and structurally diverse group of plant secondary metabolites [9]. These compounds include cyclic and acyclic 30-carbon precursors and are ubiquitously distributed in nature. Among triterpenoids, triterpenes with pentacyclic skeletons, such as oleanane, ursane, and lupane carbon skeletons, have sparked renewed interest due to their potential in cancer treatment [10,11,12]. In particular, betulinic acid (BA) and ursolic acid (UA) (Figure 1), which are abundant in the outer barks of birch trees (*Betula* spp.) and across a wide range of plant families [13], have been widely studied for their anti-tumoral activity in multiple cancer models and were found to modulate diverse pathways involved in carcinogenic processes [14,15,16,17,18,19].

Metabolic reprogramming is strongly linked to tumor-specific signaling pathways and supports tumor growth, invasion and immune escape [20]. Several drugs targeting altered metabolic enzymes and pathways in cancer are currently under intense pre-clinical and clinical testing [21]. The ability of plant-derived natural compounds to modulate tumor cell metabolism and, in this way, exert anticancer activity, has also started to be realized [8]. A few works have addressed the impact of triterpenic acids (TAs) on tumor metabolism, namely on specific glycolytic enzymes and lactate production [22,23,24,25]. However, a more comprehensive picture of their impact on both tumor and non-tumor cell metabolism is still missing.

The present work aims to assess the metabolic effects of BA and UA in MDA-MB-231 breast cancer cells (TNBC model), as well as in MCF-10A non-cancer breast epithelial cells. Identification and quantification of changes in the cells exo- and endometabolome were performed through ^1^H NMR analysis of cell culture medium supernatants, aqueous and organic cell extracts. This approach is expected to provide new insights into the involvement of metabolic reprogramming in cellular responses to these TAs, and will hopefully contribute to advance research on phytochemical-based therapy for TNBC.

## 2. Materials and Methods

### 2.1. Materials

Dulbecco’s modified Eagle’s medium (DMEM), DMEM/F12 medium and trypsin (5 g/L)-EDTA (2 g/L) were supplied by Biowest, (Nuaillé, France). Fetal bovine serum (FBS) was from Gibco (Thermo Fisher Scientific, Waltham, MA, USA). Horse serum, human epidermal growth factor, human insulin, hydrocortisone and cholera toxin were obtained from Sigma-Aldrich (St. Louis, MO, USA). Betulinic acid (≥90% purity) and ursolic acid (≥98% purity) were purchased from Molekula GmbH (Munchen, Germany). Dimethylsulfoxide (DMSO, cell culture grade) was obtained from Applichem (Gatersleben, Germany). 3-(4,5-dimethylthiazol-2-yl)-2,5-diphenyltetrazolium bromide (MTT) and propidium iodide were purchased from Calbiochem (San Diego, CA, USA). Methanol was obtained from Merk (Darmstadt, Germany) and chloroform from Normapur (VWR, Radnor, USA). RNase was obtained from Sigma Chemicals Co. (Madrid, Spain). Deuterated water (D_2_O) containing 0.75% 3-(trimethylsilyl)propionic-2,2,3,3-d_4_ acid sodium salt (TSP-d_4_) and deuterated chloroform containing 0.03% (*v*/*v*) tetramethylsilane (TMS) were obtained from Sigma-Aldrich (St. Louis, MO, USA).

### 2.2. Cell Culture

The human breast cancer cell line MDA-MB-231 and the immortalized normal breast epithelial cell line MCF-10A were purchased from American Type Cell Culture (ATCC, Manassas, VA, USA). MDA-MB-231 cells were cultured in DMEM supplemented with 10% (*v*/*v*) heat-inactivated FBS. MCF-10A cells were cultured in DMEM/F12 medium, supplemented with 5% (*v*/*v*) heat-inactivated horse serum, human epidermal growth factor (20 ng/mL), human insulin (10 μg/mL), hydrocortisone (100 ng/mL) and cholera toxin (0.1 nM). Both cell lines were cultured at 37 °C in a 5% CO_2_ humidified atmosphere (C150, Binder GmbH, Tuttlingen, Germany). After reaching subconfluence, cells were trypsinized with a trypsin (0.5 g/L) / EDTA (0.2 g/L) solution and suspended in fresh growth medium before platting. All experiments were performed during the linear phase of cellular growth.

### 2.3. Cell Viability Assay

Cells were cultured in 96-well plates at 2 × 10^5^ cells/mL (100 µL per well) and treated the following day with either BA or UA (0.5–50 µM), for 24 h, 48 h and 72 h. Vehicle solvent control cells received DMSO (0.25% *v*/*v*). Cell viability was estimated by the 3-(4,5-dimethylthiazol-2-yl)-2,5-diphenyltetrazolium bromide (MTT) assay as previously described by [26]. After treatment incubation, 20 μL of MTT stock solution was added to each well (final concentration 0.5 mg/mL), and plates were incubated at 37 °C, for 4 h. Then, the medium was removed, and the formed formazan crystals were dissolved using a DMSO/ethanol (1:1) solution. Finally, absorbance of the formed product was measured at 570 nm using a microplate reader (MultiSkan FC, Thermo Fisher Scientific, Rochester, NY, USA). The IC_50_, defined as the concentration necessary to cause 50% inhibition of cell viability, was calculated using GraphPad Prism 5.0 (GraphPad Prism Software Inc., San Diego, CA, USA), by plotting the percentage of cell viability as a function of sample concentration logarithm. Three independent experiments were performed for each treatment.

### 2.4. Cell Cycle Analysis by Flow Cytometry

MDA-MB-231 and MCF-10A cells were seeded in six-well plates at a density of 4 × 10^5^ cells/mL (2 mL per well) and cultured for 24 h at 37 °C. This was followed by 48 h incubations with BA or UA, at concentrations close to the IC_50_ values (15 and 20 µM, for BA and UA, respectively). Vehicle solvent control cells received DMSO (0.10% *v*/*v*). Then, cells were trypsinized, collected, washed with phosphate buffered saline (PBS) and fixed with 85% cold ethanol. At the time of analysis, cells were centrifuged at 300× *g* for 5 min at 4 °C and resuspended in PBS, before being treated with RNase (50 µg/mL) and propidium iodide staining solution (50 µg/mL) and incubated, in the dark, for at least 20 min at room temperature. Propidium iodide-stained cells were analyzed on a Coulter EPICS XL (Beckman Coulter, Hialeah, FL, USA) flow cytometer. The results were acquired using the SYSTEM II software (version 3.0 Beckman-Coulter ^®^, Brea, CA, USA). Four replicates were performed for each treatment, and for each sample at least 5000 nuclei were acquired. Analysis of cell cycle distribution was performed using the FlowJo software (Tree Star, Ashland, USA).

### 2.5. Cell Exposure for Metabolomics Assays

MDA-MB-231 and MCF-10A cells were seeded in 10-cm-diameter Petri dishes at a density of 6 × 10^5^ cells/mL (10 mL per dish) and cultured for 24 h at 37 °C. Cells were then incubated for 48 h with BA (5 and 15 µM) or UA (10 and 20 µM). Vehicle solvent control cells received DMSO (0.10% *v*/*v*). Samples were collected immediately after 48 h incubation or, to assess cellular recovery, medium was replaced with fresh growth medium (without TAs), and cells were incubated for additional 24 h before collection (48 + 24 h samples). Four independent assays were performed.

### 2.6. Sample Collection and Preparation for NMR Analysis

After incubation, culture medium was collected and centrifuged (1000× *g*, 5 min) and the supernatant stored at −80 °C until analysis. Culture medium without cells was placed under the same conditions and collected. Cell extraction was performed as described by Carrola et al. [27]. Briefly, cells were washed four times with PBS and extracted with a mixture of methanol:chloroform:water (1:1:0.7). The resulting polar and organic phases were collected and then dried under vacuum or under a stream of nitrogen gas, respectively. All samples were stored at −80 °C and, at the time of analysis, dried polar extracts were reconstituted in 600 µL of deuterated phosphate buffer (100 mM, pH 7.4) containing 0.1 mM TSP-d_4_, while organic extracts were reconstituted in deuterated chloroform containing 0.03% TMS. Regarding medium samples, 540 μL were mixed with 60 μL of D_2_O containing 0.25% TSP-d_4_. Prior to analysis, 550 μL of each sample were transferred to 5 mm NMR tubes.

### 2.7. NMR Data Acquisition and Analysis

^1^H NMR spectra of all samples were acquired on a Bruker Avance III HD 500 NMR spectrometer (University of Aveiro, Portuguese NMR Network) operating at 500.13 MHz for ^1^H observation equipped with a 5 mm TXI probe. Standard 1D spectra (Bruker pulse programs ‘noesypr1d’, with water suppression, for medium samples and aqueous extracts, and ‘zg’ for organic extracts) were recorded with a 7002.8 Hz spectral width, 32 k data points, a 2 s relaxation delay and 512 scans. Spectral processing comprised exponential multiplication with 0.3 Hz line broadening, zero filling to 64 k data points, manual phasing, baseline correction, and chemical shift calibration to the TSP or TMS signal at 0 ppm. 2D ^1^H-^1^H total correlation (TOCSY) spectra, ^1^H-^13^C heteronuclear single quantum correlation (HSQC) spectra and J-resolved spectra were also registered for selected samples to assist spectral assignment. Metabolites were identified with the support of 2D spectra and the spectral reference databases BBIOREFCODE-2-0-0 (Bruker Biospin, Rheinstetten, Germany) and HMDB [28]. Selected signals were then integrated using the AmixViewer software 3.9.15 (version 3.9.14, Bruker BioSpin, Rheinstetten, Germany) and normalized by the total spectral area. The magnitude of each metabolite change was assessed through the percentage of variation (and its respective error) in exposed samples relatively to controls, and through the effect size (ES) adjusted for small sample numbers (and respective standard error) [29]. Metabolite variations with absolute ES greater than 0.8 were expressed in a heatmap, colored as a function of the percentage of variation, employing the R statistical software.

### 2.8. Statistical Analysis

Cell viability and cell cycle results were analyzed using the PROC GLM option of SAS (SAS Institute Inc., Cary, NC, USA). Where differences existed, the source of the differences at *p* < 0.05 significance level was identified by all pairwise multiple comparison procedures via the Tukey’s test.

## 3. Results

### 3.1. Inhibitory Effects of Betulinic and Ursolic Acids on MDA-MB-231 and MCF-10A Cellular Viability

MDA-MB-231 and MCF-10A cells were treated with various concentrations (0–50 µM) of either BA or UA for 24 h, 48 h and 72 h, and cell viability assessed through the MTT assay. The IC_50_ values obtained are presented in Table 1. The effect of BA on MDA-MB-231 cancer cells was highly dependent on incubation time, the IC_50_ value being significantly higher for the shortest incubation period (24 h). The IC_50_ value for 48 h was 13.20 ± 2.30 µM; hence, a concentration of 15 µM and a lower concentration of 5 µM were chosen for subsequent assays. The impact of UA on MDA-MB-231 cells was less dependent on incubation time. Based on the IC_50_ value for 48 h (17.21 ± 0.86 µM), concentrations of 20 and 10 µM were selected for subsequent assays.

As for MCF-10A normal epithelial cells, they were more susceptible to BA than cancer cells, as seen by the lower IC_50_ values determined for all incubation periods (Table 1). This was not observed in the case of UA, for which the 48 h IC_50_ in MCF-10A cells (18.68 ± 3.27 µM) was similar to that found in cancer cells. In general, both cell lines were more sensitive to BA than to UA.

### 3.2. Effect of Betulinic and Ursolic Acids on MDA-MB-231 and MCF-10A Cell Cycle

**Figure 2:**
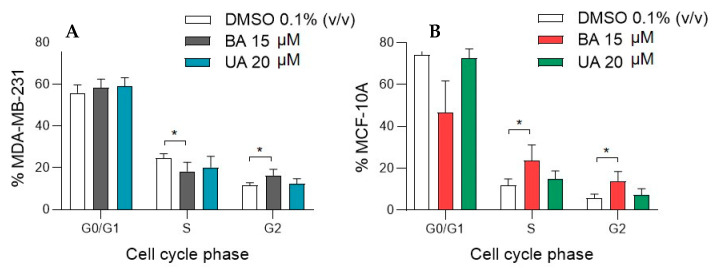
Cell cycle phase distribution of (**A**) MDA-MB-231 and (**B**) MCF-10A cells, treated with betulinic acid (BA) or ursolic acid (UA), for 48 h. DMSO was the solvent control. Each column and bar represent, respectively, the mean and the standard deviation. Four replicates considered. Statistically significant differences between DMSO controls and BA or UA treated samples are indicated with * (*p*-value < 0.05, Tukey’s test).

The effects of BA and UA on cell cycle phases (G0/G1, S and G2) of MDA-MB-231 and MCF-10A cells, as assessed by flow cytometry, are shown in Figure 2. Betulinic acid (15 µM) led to a significant accumulation of MDA-MB-231 cells at the G2 phase, together with a decreased cell population at the S phase, in comparison with non-exposed control cells. MCF-10A breast epithelial cells treated with BA also displayed a statistically significance cell cycle arrest at the G2 phase and an increase in the S phase, with a concomitant decrease in the G0/G1 phase. Ursolic acid (20 µM) had less marked effects on the cell cycle phases of both MDA-MB-231 and MCF-10A cells, with no statistical differences being noted between untreated and UA-treated cells.

### 3.3. Variations Induced by Betulinic and Ursolic Acids in the Metabolome of MDA-MB-231 and MCF-10 Breast Cells

Metabolic alterations induced by BA and UA on MDA-MB-231 breast cancer and MCF-10A breast epithelial cells were assessed immediately after 48 h incubations, as well as after a 24 h recovery period in fresh growth medium (without TAs). NMR analysis of culture medium supernatants allowed the cells exometabolome to be characterized, while intracellular metabolic variations were assessed through analysis of aqueous and organic cell extracts.

#### 3.3.1. Extracellular Metabolic Changes Induced by Betulinic and Ursolic Acids

**Figure 3:**
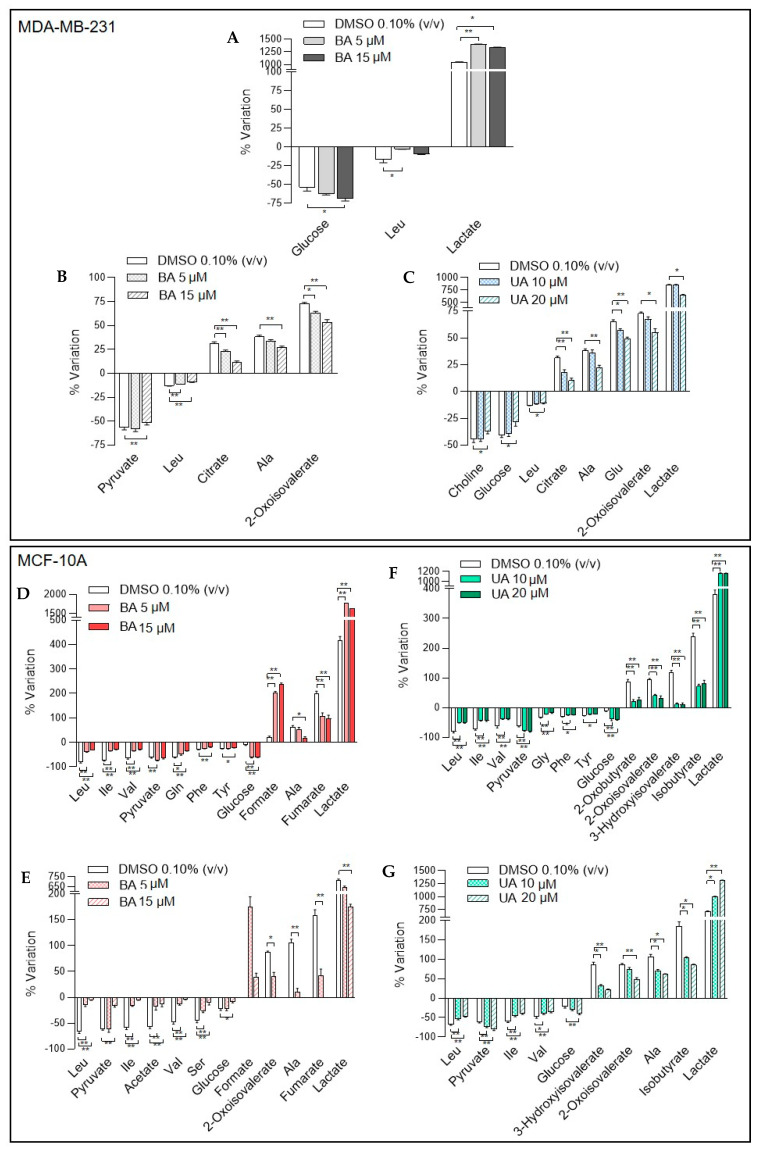
Variations in metabolites consumed (negative bars) and excreted (positive bars) by cells upon exposure to triterpenic acids (TAs), as assessed by comparison to acellular culture media. Consumption and excretion patterns are shown for control cells and: (**A**) MDA-MB-231 treated with betulinic acid (BA) for 48 h, (**B**) MDA-MB-231 treated with BA for 48 h followed by 24 h recovery in fresh medium, (**C**) MDA-MB-231 treated with ursolic acid (UA) for 48 h followed by 24 h recovery in fresh medium, (**D**) MCF-10A treated with BA for 48 h, (**E**) MCF-10A treated with BA for 48 h followed by 24 h recovery in fresh medium, (**F**) MCF-10A treated with UA for 48 h, (**G**) MCF-10A treated with UA for 48 h followed by 24 h recovery in fresh medium. Four independent replicates considered. ** *p*-value < 0.01; * *p*-value < 0.05. Three-letter code used for amino acids. Different scale used in each graph according to variation range.

Alterations in metabolite consumption and excretion patterns upon incubation of breast cells with BA or UA were assessed by comparing metabolite levels in cell-conditioned media with those in acellular growth media. Metabolite variations presenting significant differences between control and exposed cells are shown in Figure 3.

MDA-MB-231 cells incubated for 48 h with 15 µM BA showed significant increases in glucose consumption and lactate excretion, the latter being already noticed at the lower concentration tested (5 µM). On the other hand, the consumption of leucine decreased significantly with exposure to 5 µM BA (Figure 3A). Upon medium replacement and additional 24 h incubation in fresh growth medium (without TAs), cells that had been treated with BA (48 + 24 h samples) still displayed differences in their exometabolome, as compared to control cells (Figure 3B). They consumed less leucine and pyruvate and excreted less 2-oxoisovalerate, citrate and alanine. However, there were no longer differences in glucose consumption nor lactate excretion, as compared to untreated controls. As for UA effects in the exometabolome of MDA-MB-231 cancer cells, upon 48 h of incubation, no significant changes were noticed. However, when UA-treated cells recovered in fresh culture medium for 24 h, there were significant differences in several extracellular metabolites, as compared to untreated control cells (Figure 3C). Changes comprised decreased consumption of glucose, leucine and choline, accompanied by decreased excretion of lactate, 2-oxoisovalerate, citrate, alanine and glutamate.

The exometabolome of MCF-10A epithelial cells was greatly affected by incubation with either BA or UA. Compared to untreated controls, BA-treated cells significantly reduced the consumption of several amino acids, namely branched chain amino acids, glutamine, phenylalanine and tyrosine (Figure 3D). This was accompanied by decreased excretion of alanine and fumarate. On the other hand, these cells consumed more glucose and pyruvate, while excreting more lactate and formate. Upon incubation for an additional 24 h in fresh culture medium, MCF-10A cells pre-treated with BA maintained decreased consumption of some amino acids (BCAA, serine) and decreased excretion of alanine and fumarate, in relation to controls (Figure 3E). However, the consumption of glucose, pyruvate and acetate decreased, and so was the excretion of lactate and 2-oxoisovalerate. On the other hand, formate excretion increased in BA pre-treated cells (48 + 24 h samples). Regarding MCF-10A cells treated with UA for 48 h (Figure 3F), they decreased the consumption of several amino acids and the excretion of some of their metabolic products (e.g., 2-oxoisovalerate, 3-hydroxyisovalerate), while increasing the consumption of glucose and pyruvate and the excretion of lactate, similarly to BA-treated cells. Most of these changes were maintained in UA-treated cells after a 24 h recovery period (Figure 3G).

#### 3.3.2. Changes in Intracellular Polar Metabolites Induced by Betulinic and Ursolic Acids

**Figure 4:**
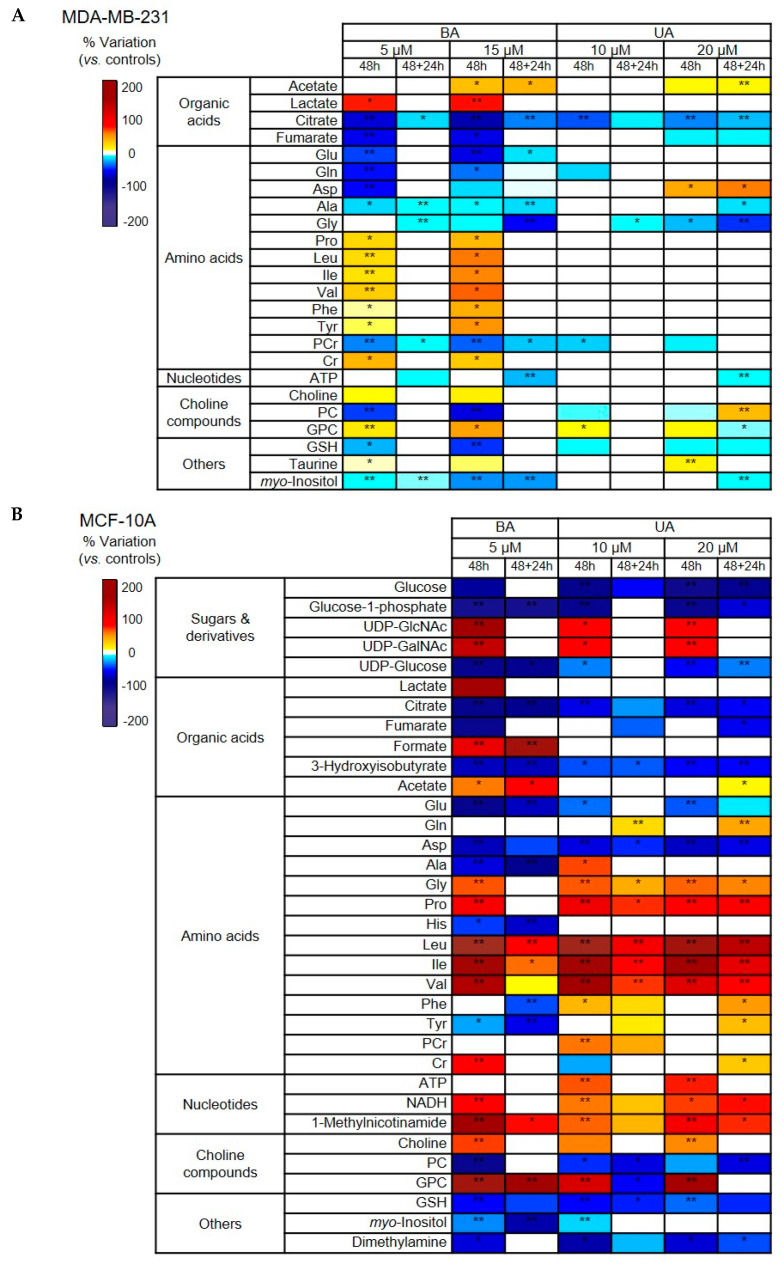
Heatmap of the main metabolite variations in polar extracts of (**A**) MDA-MB-231 cells and (**B**) MCF-10A cells, exposed to betulinic acid (BA) or ursolic acid (UA), colored according to % variation in relation to controls. Four independent replicates considered. * *p*-value < 0.05, ** *p*-value < 0.01. Three letter code used for amino acids; Cr, creatine; PCr, phosphocreatine; ATP, adenosine triphosphate; NADH, nicotinamide adenine dinucleotide (reduced); GSH, reduced glutathione; PC, phosphocholine; GPC, glycerophosphocholine; UDP, uridine diphosphate; UDP-GalNAc, UDP-N-acetyl-galactosamine; UDP-GlcNAc, UDP-N-acetyl-glucosamine.

Analysis of ^1^H NMR spectra from cellular aqueous extracts enabled the identification of 60 intracellular metabolites based on matching 1D and 2D NMR sample data to spectral data recorded in-house for standard compounds and/or deposited in other available databases (Figure S1 and Table S1 in Supplementary Information). Then, spectral integration of individual metabolites enabled relevant variations to be highlighted. The results are summarized in the form of heatmaps, color-coded according to the percentage of variation relatively to respective controls (Figure 4) and presented in full in Supplementary Tables S2 and S3 for MDA-MB-231 and MCF-10A cells, respectively.

Regarding the metabolic effects of TAs on MDA-MB-231 breast cancer cells (Figure 4A), BA had a much larger impact on the intracellular polar metabolome than UA, as seen by consistent alterations in 23 vs. 10 metabolites in cells treated with BA vs. UA for 48 h, at concentrations of 15 µM and 20 µM, respectively. In the case of BA, only a small number of changes remained (7 out of 23) or newly appeared (decrease in ATP) in cells that recovered in fresh growth medium after treatment. On the other hand, cells treated with the higher UA concentration kept their changed metabolic profile even after the 24 h recovery period and even displayed additional differences relatively to controls.

Describing the results in more detail, the intracellular metabolic changes arising from 48 h incubation of MDA-MB-231 cells with BA (5 or 15 µM) were: i) increases in acetate and lactate, some amino acids (proline, branched chain and aromatic amino acids), creatine, choline, glycerophosphocholine (GPC) and taurine; and ii) decreases in citrate and fumarate, another set of amino acids (glutamine, glutamate, aspartate, alanine and glycine), phosphocholine (PC), glutathione (GSH) and myo-inositol. As for the UA intracellular metabolic effects, they comprised: i) increases in acetate, aspartate, GPC and taurine; and ii) decreases in citrate, fumarate, glycine, phosphocreatine, PC and GSH. Interestingly, the variations in PC and GPC were inverted when cells treated with the high BA concentration were incubated in fresh medium (48 + 24 h samples). Furthermore, decreased ATP levels were only noticed in 48 + 24 h samples, as observed for BA.

The metabolic impact of TAs on MCF-10A breast epithelial cells was clearly greater than in cancer cells (at the same exposure concentrations), as seen by the more intense coloring of the respective heatmap (Figure 4B). For BA, only the low concentration (5 µM) samples were included in this analysis because the spectra obtained for cells treated with BA 15 µM were considerably noisier than all others. In total, incubation with BA (5 µM) for 48 h altered the intracellular levels of 31 polar metabolites. Out of these, 18 metabolites kept altered levels in 48 + 24 h samples. As for 48 h UA-treated epithelial cells, they showed alterations in 27/22 metabolites (at low/high UA concentration). Out of these, 17 compounds maintained their variation in cells allowed to recover in UA-free medium (48 + 24 h samples), while 4/6 new variations emerged in these cells (after low/high UA pre-treatment).

Looking closer at BA effects in MCF-10A cells, they comprised: i) increases in uridine diphosphate N-acetyl glucosamine and galactosamine (UDP-GlcNAc and UDP-GalNAc), lactate, formate, acetate, some amino acids (glycine, proline and branched chain amino acids), creatine, NADH, 1-methylnicotinamide, choline and GPC; along with ii) decreases in glucose, glucose-1-phosphate and UDP-glucose, citrate and fumarate, 3-hydroxyisobutyrate, other amino acids (glutamate, aspartate, histidine and tyrosine), phosphocholine, glutathione (GSH), myo-inositol and dimethylamine. Most of the above-mentioned variations were also found in UA-treated epithelial cells. Exceptions were seen for i) lactate, formate and histidine (no change in UA-exposed cells), ii) glutamine, ATP and phosphocreatine (variations detected in UA but not in BA-treated cells), and iii) alanine, phenylalanine and tyrosine (opposite variations in BA and UA-exposed cells).

#### 3.3.3. Changes in Lipid Composition Induced by Betulinic and Ursolic Acids

**Figure 5:**
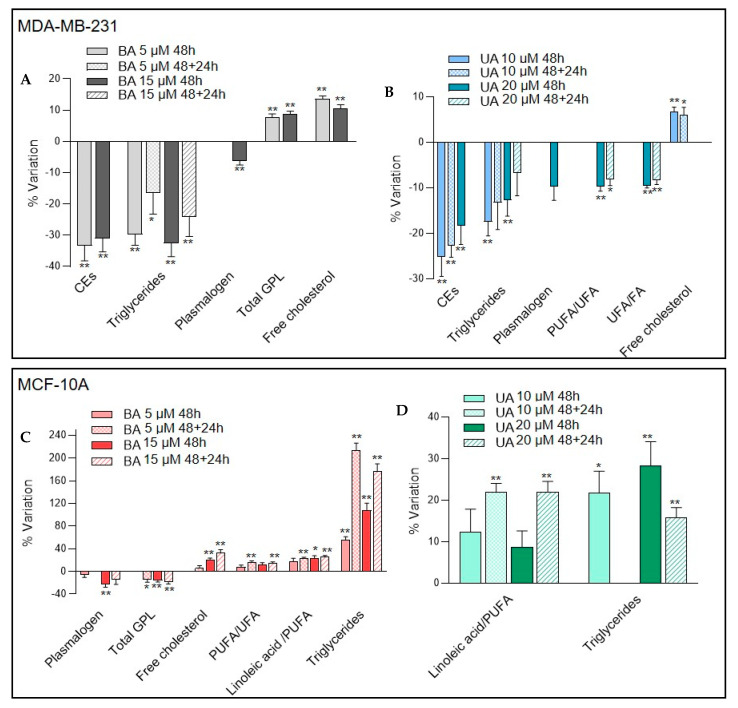
Lipid-related variations in organic extracts from MDA-MB-231 breast cancer cells (**A**,**B**) and MCF-10A cells (**C**,**D**), treated with betulinic acid (BA) or ursolic acid (UA), relatively to untreated controls. Four independent replicates considered. ** *p*-value < 0.01; * *p*-value < 0.05. CEs, cholesteryl esters; PUFA, polyunsaturated fatty acyl chains, UFA, unsaturated fatty acyl chains; GPL, Glycerophospholipids; FA, total fatty acids. Different scale used in each graph according to variation range.

NMR analysis of cells organic extracts enabled the detection and quantitative assessment of several lipid species, such as cholesterol (free and esterified), triglycerides and phospholipids (Figure S2 and Table S4 in Supplementary Information). Furthermore, the signals of BA and UA were clearly detected in the 1D ^1^H NMR spectra of exposed cells (Figure S2), confirming their direct interaction with both MDA-MB-231 cancer cells and MCF-10A epithelial cells, either through internalization and/or membrane association. The changes in cellular lipid components were then assessed through spectral integration and normalization of signal areas to the total spectral area, excluding both residual solvent signals and the TA resonances identified. The significant variations obtained for MDA-MB-231 and MCF-10A cells are presented in Figure 5.

When MDA-MB-231 breast cancer cells were incubated with BA for 48 h, the levels of neutral lipids (cholesteryl esters and triglycerides) decreased significantly relatively to control cells (Figure 5A). Plasmalogen lipids (glycerophospholipids with a vinyl–ether bond at the sn-1 position) also decreased, at the high exposure concentration only, while total glycerophospholipids and free cholesterol increased in BA-treated cells. However, except for triglyceride reduced levels, all other changes were reversed in BA-treated cells that were incubated for additional 24 h in BA-free fresh medium. As found for BA, 48 h cellular incubations with UA (Figure 5B) resulted in decreased levels of plasmalogen (high concentration), cholesteryl esters and triglycerides (both concentrations), along with an increase in free cholesterol (seen only at the low exposure concentration). However, contrarily to BA, most changes occurring in 48 h UA-treated cells persisted after the 24 h UA-free incubation period. In addition, cancer cells treated with 20 µM UA displayed a reduced relative proportion of unsaturated (namely, polyunsaturated) fatty acyl chains composing cellular lipids.

Compared to MDA-MB-231 cells, MCF-10A cells displayed a very different profile of lipid variations upon incubation with TAs. As shown in Figure 5C, BA-treated epithelial cells displayed decreased levels of glycerophospholipids (including plasmalogen) and increased levels of triglycerides. The relative amount of free cholesterol was also increased, and so was the contribution of polyunsaturated fatty acids (such as linoleic acid) to the total fatty acyl content. All changes persisted after the 24 h recovery period. As for UA-treated MCF-10A cells, significant variations in comparison to control cells were confined to increases in triglycerides and the linoleic/PUFA ratio (Figure 5D). The latter change was more pronounced in 48 + 24 h samples.

## 4. Discussion

In this work, TNBC cells (MDA-MB-231) and non-malignant breast epithelial cells (MCF-10A) were incubated with two pentacyclic triterpenes—betulinic acid (BA) and ursolic acid (UA)—with anticancer activity. The results of the MTT assay, which reflect overall cellular metabolic activity and viability [30], indicated that both cell lines were susceptible to BA and UA, in a dose- and time-dependent manner. The IC_50_ values determined for 24 h incubations of MDA-MB-231 cells with BA (31.28 µM) and UA (24.54 µM) were very similar to those recently reported in the literature (30.6 and 22.9 µM, for BA and UA, respectively) [25]. For this incubation period, UA appeared to be more cytotoxic than BA. However, for longer incubations (48 and 72 h) of MDA-MB-231 cells with each of these TAs, BA had a higher impact on MTT-assessed metabolic activity, and the respective IC_50_ values were lower than those determined for UA. Regarding MCF-10A epithelial cells, they were more susceptible to BA treatment than cancer cells (lower IC_50_ values for all incubation periods). This is in contrast with previous studies where BA was reported to have no influence on MCF-10A cells viability [31,32]. In the case of UA, we have found IC_50_ values in MCF-10A similar to those determined in MDA-MB-231 cells, while lower effects were also reported in the literature [33,34,35,36]. Differences in cell densities and incubation periods could possibly justify these discrepancies. It should be noted that, in the present study, we used the same cell density for cancer and non-cancer cells, to avoid underestimation of cytotoxic effects. Regrettably, as is often observed with chemotherapy drugs, the two TAs tested were found to be equally or slightly more cytotoxic to non-cancer than to cancer cells. This underlines the importance of developing tumor-selective drug delivery systems for these compounds, as successfully exploited for other plant-derived anticancer drugs [37].

The main goal of this study was to characterize the changes induced by BA and UA in the metabolome of both cancer (MDA-MB-231) and non-cancer (MCF-10A) breast cells, as a means to improve current knowledge of their modes of action at the molecular level. For that purpose, untargeted NMR metabolomics was employed to assess metabolic changes upon 48 h incubations with either BA (5 and 15 µM) or UA (10 and 20 µM), the higher concentrations tested corresponding approximately to the IC_50_ values. Moreover, the metabolic profiles of BA/UA-treated cells incubated for additional 24 h in fresh growth media (without TAs) were also characterized to assess the persistence/reversibility of changes. The integrated analysis of variations in cell consumption and excretion patterns, polar intracellular metabolites and lipid composition, compared to adequate controls, allowed several hypotheses on BA/UA-induced metabolic modulation of each cell type to be proposed, as discussed below.

Increased glucose consumption and lactate excretion, together with increased intracellular lactate levels, were observed upon 48 h BA treatment of either MDA-MB-231 or MCF-10A cells, compared to respective controls. These variations suggest increased flux through glycolysis, which is somewhat unexpected, given the well-established link between glycolytic upregulation and malignant progression [20]. However, the transient nature of these BA-induced effects should be noted. After 24 h in fresh growth medium, MDA-MB-231 cells previously incubated with BA (48+24 h samples) no longer showed altered glycolytic activity, and MCF-10A cells even showed reduced glucose consumption and lactate excretion compared to controls. In the literature, there are discrepant results concerning the modulation of breast cancer cell glycolytic activity by anti-cancer agents. MDA-MB-231 cells treated with common chemotherapy drugs (cisplatin and doxorubicin) have been reported to increase lactate content, hence, glycolytic activity [38]. However, in another study, a 24 h treatment of MDA-MB-231 cells with BA (up to 20 µM) did not alter lactate levels nor the expression of glycolytic enzymes [25], while BA-treated SK-BR-3 and MCF-7 breast cancer cells displayed decreased protein levels of hexokinase (HK2) and/or pyruvate kinase PKM2 [24,25]. These different results illustrate the dependence of metabolic modulation on the specific cell line considered, as well on other factors such as exposure time and concentration. Regarding UA, it appeared to differentially affect glucose metabolism in cancer and non-cancer breast cells. MDA-MB-231 cells incubated with UA for 48 h showed unaltered glycolytic activity at the end of the exposure period and a trend for downregulated glycolysis after recovery in fresh medium, along with decreased ATP content. This is in line with the results of Lewinska and co-workers, where the expression of glycolytic enzymes (HK2 and PKM2) and ATP content decreased in MDA-MB-231 treated with UA (5–20 µM) [25]. On the other hand, in MCF-10A cells, UA appeared to persistently stimulate glucose uptake and glycolytic conversion.

**Figure 6:**
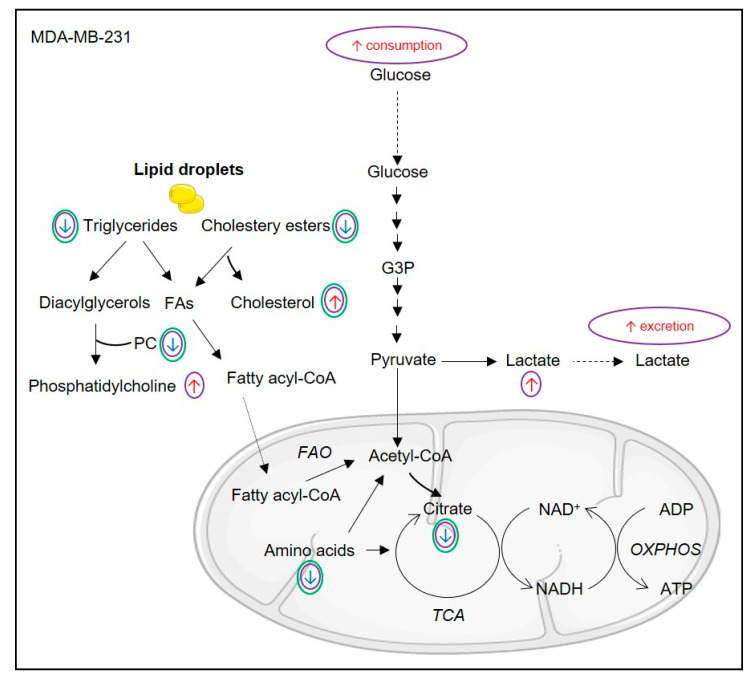
Proposed metabolic reprogramming of MDA-MB-231 breast cancer cells upon 48 h incubation with betulinic acid (BA) or ursolic acid (UA). Effects of BA and UA are represented by purple and green circles, respectively. NAD+/NADH, nicotinamide adenine dinucleotide/reduced form; PC, phosphocholine; FAs, fatty acids; TCA, tricarboxylic acid cycle; OXPHOS; Oxidative phosphorylation.

**Figure 7:**
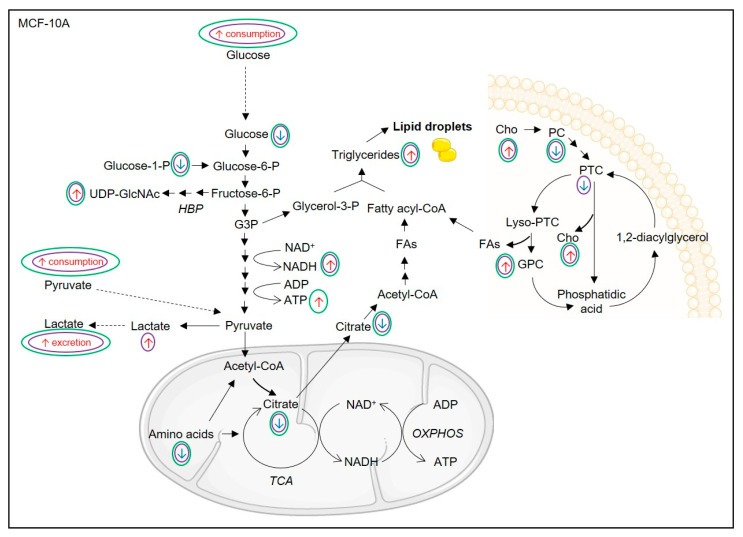
Proposed metabolic reprogramming of MCF-10A breast epithelial cells upon 48 h incubation with BA or UA. Effects of BA and UA are represented by purple and green circles, respectively. ADP/ATP, adenosine di/triphosphate; NAD+/NADH, nicotinamide adenine dinucleotide/reduced form; Cho, choline; PC, phosphocholine; GPC, glycerophosphocholine; PTC, phosphatidylcholine; UDP, uridine diphosphate; UDP-GlcNAc, UDP-N-acetyl-glucosamine; FAs, fatty acids; HBP, hexosamine biosynthetic pathway; TCA, tricarboxylic acid cycle; G3P, glyceraldehyde 3-phosphate; OXPHOS; Oxidative phosphorylation.

Changes in the levels of citrate and fumarate are indicative of tricarboxylic acid (TCA) cycle modulation. BA- and UA-treated MDA-MB-231 cells showed decreased intracellular amounts of these organic acids (Figure 6). Moreover, several amino acids that may serve as TCA cycle anaplerotic substrates (glutamate, glutamine, aspartate, alanine and glycine) decreased (especially upon BA treatment), thus suggesting enhanced flux through this metabolic pathway [39]. This effect was also apparent in BA/UA-treated MCF-10A cells, which additionally showed increased pyruvate consumption from the extracellular medium (Figure 7). On the other hand, several other amino acids displayed increased levels in treated cells, which could possibly reflect autophagic protein degradation, as previously observed in breast cells in response to nutrient deprivation [40]. Such increases, seen mostly for branched chain and aromatic amino acids, were especially pronounced in BA/UA-treated non-cancer cells, being milder in BA-treated MDA-MB-231 cells and absent in cancer cells incubated with UA.

Our results further showed a significant decrease in glutathione (GSH) upon incubation of MDA-MB-231 cells with BA and of MCF-10A cells with either BA or UA. The crucial role of this antioxidant tripeptide in neutralizing reactive oxygen species (ROS) and sustaining the survival of TNBC cells has been previously reported [41]. Hence, decreased levels of GSH may reflect increased production of ROS, which agrees with the redox imbalance previously reported to arise from exposure of breast cells to terpenic acids [25]. When moderately and/or transiently heightened, ROS can trigger the activation of adaptive mechanisms, such as the stimulation of glucose uptake, which in turn contributes to regulate the balance between ROS generation and scavenging [42]. This interplay possibly explains the upregulated glucose consumption observed herein. Notably, BA/UA-treated MCF-10A cells additionally showed accumulation of UDP-N-acetyl glucosamine/galactosamine, which are end products of the hexosamine biosynthetic pathway (HBP) and substrates in protein glycosylation. This could also relate to ROS production, as excessive ROS may inhibit the glycolytic enzyme glyceraldehyde-3-phosphate dehydrogenase (GAPDH) and cause upstream intermediates to be channeled into alternative pathways such as the HBP [43].

The levels of choline-containing metabolites, which are involved in phospholipid metabolism and associated with breast cancer progression [44], were also significantly affected in breast cells incubated with BA/UA. NMR analysis of organic cell extracts provided further insights into lipid alterations. MDA-MB-231 cells treated with TAs displayed significantly increased levels of free cholesterol and, in the case of BA, of glycerophospholipids (GPL), which are major membrane constituents. On the other hand, triglycerides and cholesteryl esters decreased upon BA/UA treatment (Figure 6). These neutral lipids are typically found in cytosolic lipid droplets, which are abundant in cancer cells and associated with their survival [45]. Interestingly, in breast cancer tissue, the intratumor accumulation of cholesteryl esters has been linked to high proliferation and aggressiveness [46]. Lipid metabolism reprogramming observed in cancer cells possibly relates to the cell cycle results, namely the observed increase at the G2 phase, during which cells enhance phospholipid synthesis to prepare for the mitotic phase [47]. This observation is also in agreement with the previously reported cell cycle arrest at G2/M phase in MDA-MB-231 cells treated with BA (2.5–10 μM) for 48 h [48]. Interestingly, the effects of TAs on lipid composition of non-cancer MCF-10A cells were drastically different from those observed in MDA-MB-231 cells. Treatment of epithelial breast cells with BA induced a decrease in glycerophospholipids, together with prominent accumulation of triglycerides and free cholesterol (Figure 7). The effects of UA were milder, but a significant increase in triglycerides was also noted. This could reflect the diversion of glycerol-3-phosphate from glycolysis to diacylglycerol synthesis, due to the abovementioned inhibition of GAPDH [43]. The formation of cytosolic lipid droplets (LD) incorporating neutral lipids is a common adaptation to cellular stress triggered by factors such as redox imbalance, excessive free fatty acids or nutrient starvation [45,49]. Indeed, LD are currently recognized to function not only as energy reserves, but also as storage sites for otherwise-harmful lipids or proteins and as regulators of lipid homeostasis in membranes. The observed increase in PUFA, especially linoleic acid, is also consistent with their sequestration from membranes into LDs, where they are thought to be less susceptible to peroxidation reactions [50].

The metabolism of MDA-MB-231 cells was differentially affected by the two TAs tested, with BA having a stronger impact on cellular metabolic pathways. Overall, the main BA-induced alterations suggested a transient increase in glucose consumption and glycolytic conversion, possibly stimulated by ROS, together with intensification of the TCA cycle and degradation of lipid droplets. On the other hand, in MCF-10A breast epithelial cells, the metabolic signatures of BA and UA were broadly similar. The main effects comprised reprogramming of glucose metabolism, involving heightened glucose consumption and channeling of glycolytic intermediates to the HBP and the synthesis of neutral lipids. Additionally, breast epithelial cells intensified pyruvate consumption and TCA cycle activity, possibly to compensate for the ROS-induced impairment of pyruvate glycolytic production, which allowed ATP levels to be maintained or even increased.

The great differences observed in the metabolic responses of the two tested cell lines support the idea that metabolic pathways specifically altered in tumors may represent vulnerabilities that can potentially be targeted with minimum damage to healthy cells. However, it remains to be investigated whether the observed responses are common to other TNBC cell lines and to cell models of other breast cancer subtypes. Furthermore, while two-dimensional cell culture systems represent a practical approach in drug testing, they poorly mimic the dynamic tumor microenvironment and the metabolic crosstalk within it. Metabolomics profiling to 3D co-culture systems and of their modulation by anticancer compounds may be a potentially useful approach to tackle this limitation. Furthermore, specific gene/protein expression studies and independent measurements of metabolic parameters (e.g., oxygen consumption, glycolytic flux, mitochondrial activity) are warranted to further verify the biochemical hypotheses generated herein.

## 5. Conclusions

This untargeted metabolomics study revealed a panoply of unanticipated metabolic alterations in TNBC and non-cancer breast epithelial cells, upon incubation with pure triterpenic acids, BA and UA. By integrating the changes observed in cell-conditioned culture medium, intracellular polar extracts and organic extracts, it was possible to propose multiple metabolic pathways to be implicated in the cellular responses to TAs. The metabolome of MDA-MB-231 cells was markedly altered by BA, while UA produced milder metabolic effects in this cell line. The metabolic adaptations of MCF-10A cells upon exposure to either BA or UA were even more pronounced, which underscores a major role for metabolic reprogramming in the response of breast epithelial cells to the TAs tested. Acquaintance of these metabolic alterations may potentially be harnessed to achieve increased protection of non-cancer cells while targeting the destruction of cancer cells.

## Figures and Tables

**Table 1:** IC_50_ values regarding cell viability inhibition by betulinic acid (BA) and ursolic acid (UA) on the MDA-MB-231 and MCF-10A cell lines, as determined through the MTT assay.

	IC_50_ (µM) ^1^			
	MDA-MB-231		MCF-10A	
	BA	UA	BA	UA
**24 h**	31.28 ± 5.94 ^a,b^	24.54 ± 4.61 ^b,c^	22.42 ± 6.49 ^c^	37.87 ± 8.93 ^a^
**48 h**	13.20 ± 2.30 ^d,e,f^	17.21 ± 0.86 ^c,d,e,f^	10.99 ± 1.83 ^e,f,g^	18.68 ± 3.27 ^c,d,e^
**72 h**	8.39 ± 0.98 ^f,g^	17.70 ± 2.81 ^c,d,e^	2.38 ± 1.36 ^g^	21.49 ± 4.55 ^c,d^

^1^ Each value is expressed as mean ± standard deviation. Three independent experiments were carried out. Means marked with different letters are statistically different (*p* < 0.05).

## Supplementary Materials

The following are available online at https://www.mdpi.com/2218-273X/10/8/1163/s1, Figure S1. NMR spectra of an aqueous extract from MDA-MB-231 breast cancer cells. Expansions of: A) 1D ^1^H spectrum, B) ^1^H-^1^H TOCSY spectrum, C) ^1^H-^13^C HSQC spectrum. Figure S2. A) ^1^H NMR spectra of organic extracts collected from MDA-MB-231 control cells (black) and cells treated for 48 h with BA (blue). The ^1^H NMR spectrum of BA 1 mM in CDCl3 is represented in red. B) MDA-MB-231 control cells (black) and cells treated for 48 h with UA (blue). The ^1^H NMR spectrum of UA 1 mM in CDCl3 is represented in red. Table S1. Assignment of resonances in the NMR profile of polar extracts from MDA-MB-231 cells and MCF-10A cells. Table S2. Main metabolite variations in polar extracts of MDA-MB-231 cells exposed to 5 µM and 15 µM of BA and 10 µM and 20 µM of UA, in relation to controls, expressed as % variation (%var) and respective error (±), effect size (ES) and *p*-value (p). Table S3. Main metabolite variations in polar extracts of MCF-10A cells exposed to 5 µM and 15 µM of BA and 10 µM and 20 µM of UA, in relation to controls, expressed as % variation (%var) and respective error (±), effect size (ES) and *p*-value (p). Table S4. Assignment of resonances in the NMR profile of organic extracts from MDA-MB-231 cells and MCF-10A cells.
