# Supplementary material for: Triple Negative Breast Cancer and Breast Epithelial Cells Differentially Reprogram Glucose and Lipid Metabolism upon Treatment with Triterpenic Acids
Source: Biomolecules. 2020 Aug 8;10(8):1163. doi: 10.3390/biom10081163 (PMC7464159; doi:10.3390/biom10081163)
Supplement: Supplementary file 1 [file biomolecules-10-01163-s001.pdf]

Supplementary Information

Triple negative breast cancer and breast epithelial cells differentially reprogram glucose and lipid metabolism upon treatment with triterpenic acids

Ângela R. Guerra [1,2], Ana F. Paulino [2], Maria M. Castro [2], Helena Oliveira [3], Maria F. Duarte [2,4,*] and Iola F. Duarte [1,*]

[1]CICECO-Aveiro Institute of Materials, Department of Chemistry, University of Aveiro, 3810-193 Aveiro, Portugal.

[2]Centro de Biotecnologia Agrícola e Agro-Alimentar do Alentejo (CEBAL)/Instituto Politécnico de Beja (IPBeja), Apartado 6158, 7801-908 Beja, Portugal.

[3]Department of Biology & CESAM, University of Aveiro, 3810-193 Aveiro, Portugal.

[4]MED – Mediterranean Institute for Agriculture, Environment and Development, CEBAL, 7801-908 Beja, Portugal.

* Correspondence: fatima.duarte@cebal.pt; Tel.: + 351 284314399 (M.F.D); ioladuarte@ua.pt; Tel.: + 351 234401418 (I.F.D.)

Figure S1. NMR spectra of an aqueous extract from MDA-MB-231 breast cancer cells. Expansions of: A) 1D ^{1}H spectrum, B) ^{1}H-^{1}H TOCSY spectrum, C) ^{1}H-^{13}C HSQC spectrum. Signals are numbered according to Supplementary Table S1.

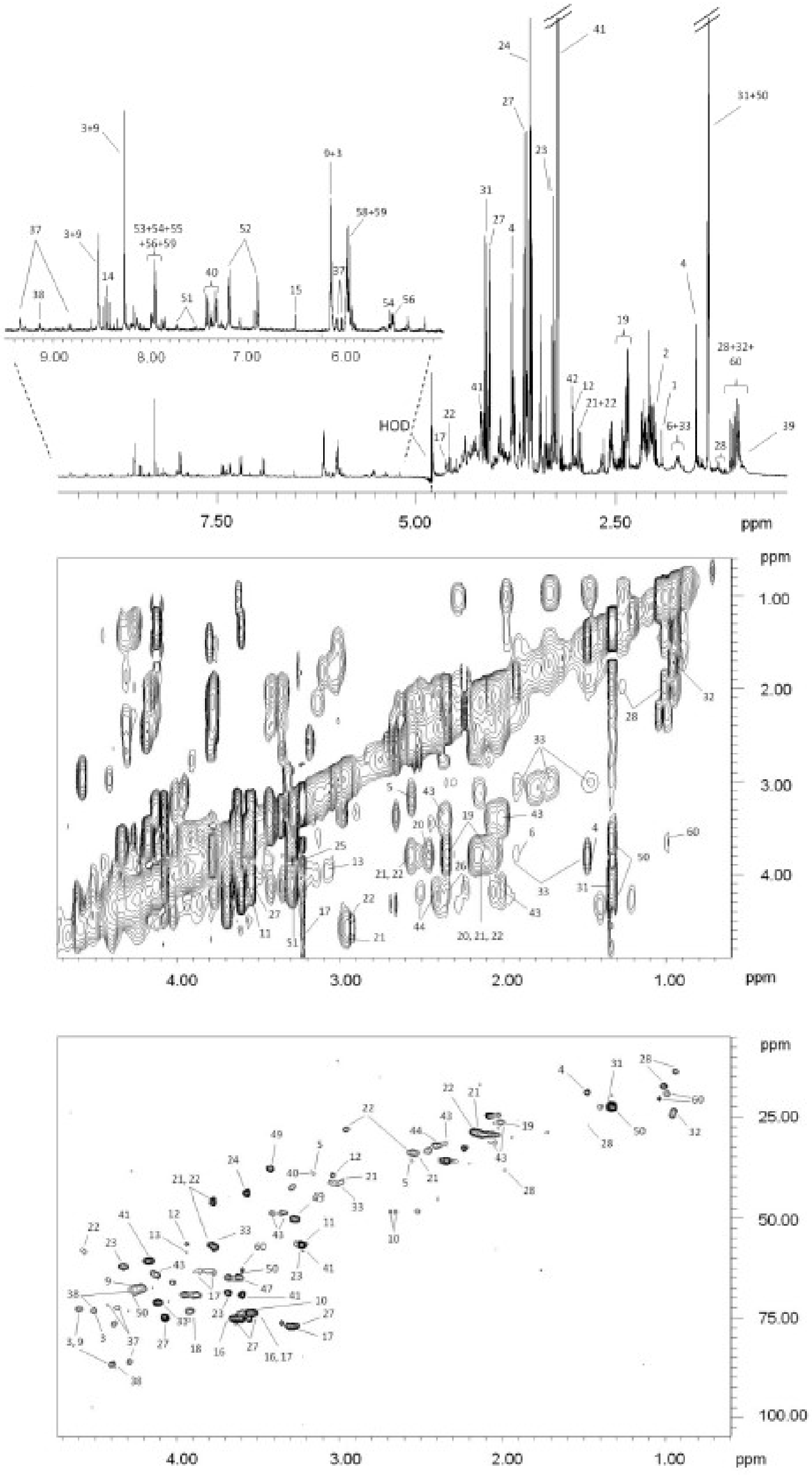

Figure S2. A) ^{1}H NMR spectra of organic extracts collected from MDA-MB-231 control cells (black) and cells treated for 48h with BA (blue). The ^{1}H NMR spectrum of BA 1mM in $CDCl_3$ is represented in red. B) MDA-MB-231 control cells (black) and cells treated for 48h with UA (blue). The ^{1}H NMR spectrum of UA 1mM in $CDCl_3$ is represented in red. The signals arising from TAs in treated cells are indicated in the respective spectra with red arrows. Lipid signals are numbered according to Supplementary Table S4.

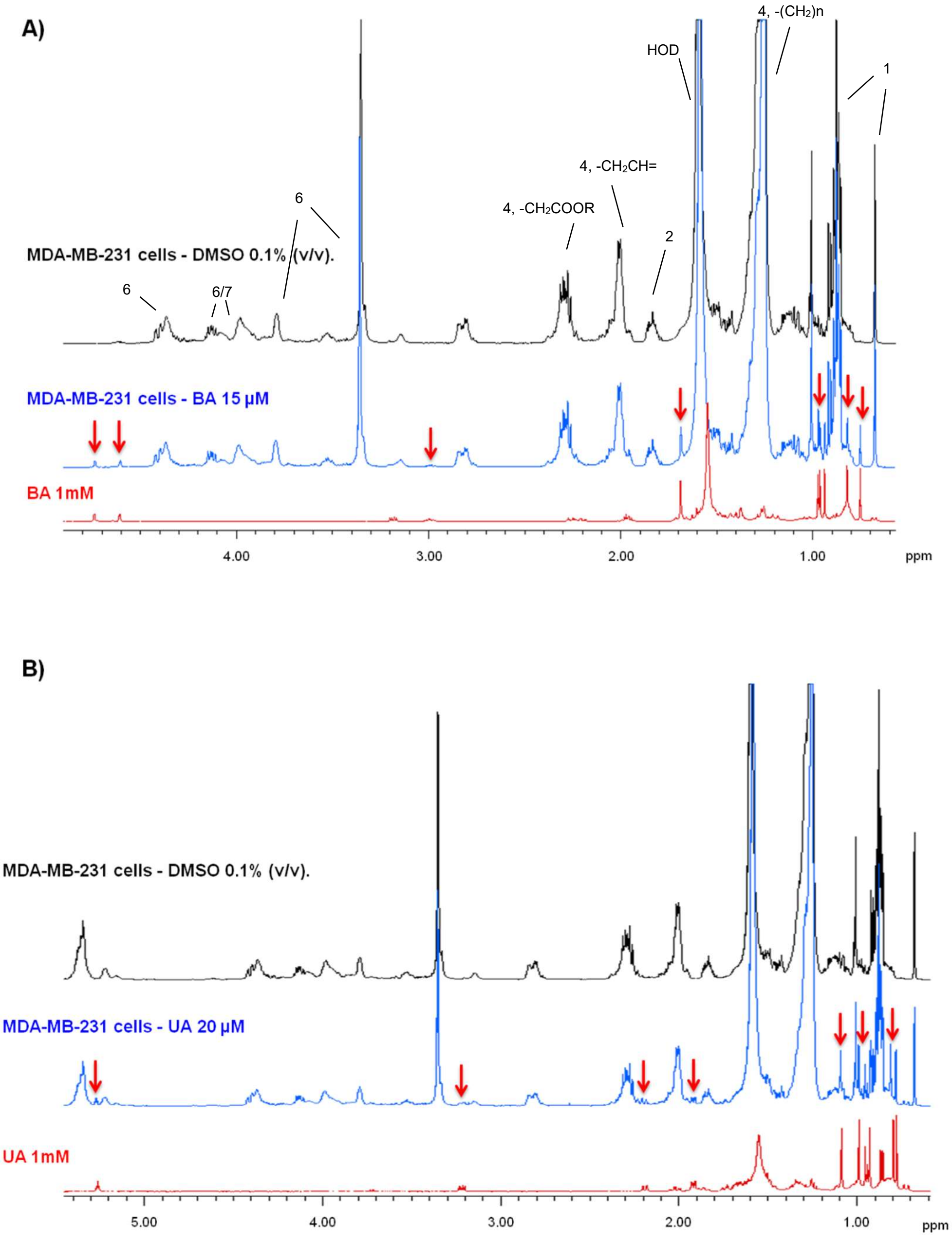

Table S1. Assignment of resonances in the NMR profile of polar extracts from MDA-MB-231 cells and MCF-10A cells. Multiplicity: s, singlet; d, doublet; t, triplet; m, multiplet; dd, double of doublets; br, broad signal.

No.	Compound	δ ^{1}H in ppm (multiplicity/ assignment)/ δ ^{13}C in ppm	Cell type	
			MDA-MB-231	MCF-10A
1	Acetate	1.92 (s; β-CH_3)	✓	✓
2	N-Acetylaspartate	2.01 (s, CH_3); 2.48 (dd, β-CH_2); 2.68 (dd, β'-CH_2); 4.38 (dd, α-CH)	✓	✓
3	ADP	4.21 (m, C5'H, ribose); 4.38 (m, C4'H, ribose)/86.25; 4.50 (m, C2'H, ribose)/72.96; 6.14 (d, C1'H, ribose)/89.49; 8.28 (s, C8, ring); 8.54(s, C2, ring)	✓	✓
4	Alanine	1.48 (d, β-CH_3)/18.47; 3.78 (q, α-CH)	✓	✓
5	β-Alanine	2.56 (t, β-CH_2)/36.00; 3.17 (t, α-CH_2)/39.18	✓	✓
6	Arginine	1.65 (m, γ-CH_2); 1.92 (m, β-CH_2); 3.23 (t, δ-CH_2); 3.76 (α-CH_3)	✓	✓
7	Asparagine	2.85 (dd, β-CH_3); 2.95 (dd, β'-CH_3); 3.99 (dd, α-CH)	✓	✓
8	Aspartate	2.70 (dd, β-CH); 2.80 (dd, β'-CH); 3.90 (dd, α-CH)	✓	✓
9	ATP	4.23 (m, C5'H, ribose)/67.34; 4.29 (m, C5''H, ribose); 4.38 (m, C4'H, ribose)/86.25; 4.59 (m, C2'H, ribose)/72.59; 6.14 (d, C1'H, ribose)/89.49;8.28 (s, C2, ring); 8.54 (s, NH, ring)	✓	✓
10	Citrate	2.53 (d, CH_3)/48.07; 2.65 (d, α', β'-CH_2)/48.33	✓	✓
11	Choline	3.21 (s, $N(CH_3)_3$)/56.53; 3.52 (m, CH_2 (NH)); 4.05 (m, CH_2(OH))	✓	✓
12	Creatine	3.03 (s, CH_3)/39.62; 3.92 (s, CH_2)/56.35	✓	✓
13	Cysteine	3.09 (m, β-CH2); 3.94 (dd, α-CH)/58.74	✓	✓
14	Formate	8.46 (s, CH)	✓	✓
15	Fumarate	6.52 (s, CH)	✓	✓
16	α-Glucose	3.41 (m, C4H); 3.54 (dd, C2H)/73.63; 3.67 (m, C3H)/75.19; 3.81 (m, C6H)/63.11; 3.85 (m, C5H)/63.11; 5.23 (d, C1H)	✓	✓
17	β-Glucose	3.28 (dd, C2H)/76.83; 3.41 (m, C4H); 3.47 (m, C5H); 3.48 (t, C3H); 3.76 (m, C6H)/63.11; 3.85 (dd C6'H)/63.11; 4.65 (d, C1H)	✓	✓

Table S1. (cont.)

No.	Compound	δ ^{1}H in ppm (multiplicity/ assignment)/ δ ^{13}C in ppm	Cell type	
			MDA-MB-231	MCF-10A
18	Glucose-1-phosphate	3.40 (t, C4H); 3.49 (m, C2H); 3.77 (m, C3H); 3.75 (m, C6H); 3.86 (m C6'H); 3.91 (m, C5'H)/73.20; 5.46 (dd, C1H)	✓	✓
19	Glutamate	2.00 (m, β-CH_2)/26.35; 2.13 (m, β'-CH_2); 2.34 (m, γ-CH_2); 3.75 (α CH);	✓	✓
20	Glutamine	2.11 (m (m, β-CH_2); 2.45 (m, γ-CH_2); 3.78 (t, α-CH)	✓	✓
21	Glutathione, oxidised (GSSG)	2.16 (m, β-CH_2 , Glu)/28.95; 2.55 (m, γ-CH_2 , Glu)/23.95; 3.00 (m, β-CH_2, Cys)/41.60; 3.30 (m, β-CH_2 , Cys')/41.15; 3.77 (α-CH, Gly)/45.97; 3.76 (α-CH, Glu)/56.85; 4.74 (m, α-CH, Cys)/55.17	✓	✓
22	Glutathione, reduced (GSH)	2.16 (m, β-CH_2, Glu)/28.83; 2.54 (m, γ-CH_2, Glu)/33.91; 2.95 (m, β-CH_2, Cys)/28.14; 3.77 (α-CH, Gly)/45.97; 3.78 (α-CH, Glu)/56.8; 4.56 (m, α-CH, Cys)/58.30; 8.35 (NH, Gly)	✓	✓
23	Glycerophosphocholine (GPC)	3.23 (s, N(CH_3)3)/56.72; 3.68 (β'-CH_2(N))/68.52; 4.32 (m, α'-CH_2(P)/61.97	✓	✓
24	Glycine	3.56 (s, α-CH_2)/43.98	✓	✓
25	Histidine	3.27 (m, β-CH_2); 3.99(dd αCH); 7.08 (s, C4H, ring); 7.84 (s, C2H, ring)	✓	✓
26	3-Hydroxybutyrate	1.08 (d, γ-CH_3); 2.28 (dd, CH_2); 2.42 (dd, CH_2); 4.14 (m, CH)	✓	✓
27	*myo*-Inositol	3.27 (t, C5H)/77.01; 3.54 (C1H, C3H)/73.71; 3.61 (dd, C4H, C6H)/75.00; 4.07 (t, C2H)/74.85	✓	✓
28	Isoleucine	0.94 (t, δ-CH_3)/13.58; 1.00 (d, β'-CH_3)/17.26; 1.27 (m, γ-CH_2)/27.33; 1.47 (m, γ'-CH_2)/27.01; 1.97 (m, β-CH)/38.33	✓	✓
29	α-Ketoglutarate	2.44 (t, β-CH_2); 3.00 (t, γ-CH_2)	✓	✓
30	Ketoleucine	0.92 (d, δ-CH_3); 2.10 (m, γ-CH); 2.61 (d, β-CH_2)	✓	✓
31	Lactate	1.32 (d, β-CH_3)/22.60; 4.10 (m, α-CH)/71.09	✓	✓
32	Leucine	0.95 (d, δ-CH_3)/23.47; 0.96 (d, δ'-CH_3)/24.61; 1.69 (m, γ-CH)/42.48;1.72 (m, β-CH 2); 3.73 (t, α-CH)	✓	✓

Table S1. (cont.)

No.	Compound	δ ^{1}H in ppm (multiplicity/ assignment)/ δ ^{13}C in ppm	Cell type	
			MDA-MB-231	MCF-10A
33	Lysine	1.49 (m, γ-CH_2); 1.72 (m, δ-CH_2)/28.86; 1.90 (m, β-CH_2); 3.00 (t, ε-CH_2)/41.57; 3.76 (t, α-CH)/57.10	✓	✓
34	Malate	2.38 (dd, β'-CH); 2.67 (dd, β-CH); 4.30 (dd, α-CH)	✓	✓
35	Methionine	2.13 (s, β-CH_2); 2.16 (t, β-CH_2); 2.61 (t, γ-CH_2); 3.85 (t, α-CH)	✓	✓
36	3-Methyl-2-oxovalerate	0.90(t, δ-CH_3), 1.10 (d, β'-CH_3), 1.45(m, γ-CH), 1.70 (m, γ'-CH_2), 2.95 (m, β-CH)	✓	✓
37	NAD^+	4.28 (m, A5')/85.91; 4.36 (m, A4')/72.35; 4.38 (m, A4'/N5'); 4.42 (dd, N3')/ 72.64; 4.50 (m, A3'); 4.55 (m, N2'); 6.04 (d, N1'); 6.09 (d, A1'); 8.18 (s, A2); 8.20 (N5); 8.43 (s, A8); 8.84 (d, N4); 9.15 (d, N6); 9.34 (s, N2)	✓	✓
38	NADH	2.70 (m, N4); 4.08 (br. s., N2'); 4.17 (m, N4'); 4.21 (d, N3'); 4.25 (m, A5')/67.70; 4.40 (m, A4')/86.32; 4.50 (m, A3')/73.18; 4.75 (N5); 5.98 (dd, N6); 6.12 (d, A1'); 6.95 (d, N2); 8.23 (s, A2), 8.48 (s, A8)	✓	✓
39	Pantothenate	0.90 (s, CH_3); 0.94 (s, CH_3); 2.42 (t, α-CH_2); 3.38 (d, CH_2); 3.43 (q, β-CH_2); 3.50 (d, CH_2); 3.98 (s, CH)	✓	✓
40	Phenlyalanine	3.16 (m, β-CH)/ 38.27; 3.29 (dd, β'-CH); 3.99 (m, α-CH)/56.67; 7.32 (d, C2H, C6H, ring)/131.95; 7.39 (d, C4H, ring); 7.41 (t, C3H, C5H, ring)/131.70	✓	✓
41	Phosphocholine (PC)	3.22 (s, $N(CH_3)3$)/56.72; 3.59 (m, N-CH_2)/69.00; 4.16 (m, PO_3 -CH_2)/60.67	✓	✓
42	Phosphocreatine	3.04 (s, CH_3); 3.95 (s, CH_2)	✓	✓
43	Proline	1.99 (m, γ-CH_2)/26.35; 2.05 (m, β-CH)/31.44; 2.34 (m, β'-CH)/31.54; 3.34 (dt, δ-CH)/48.61; 3.40 (dt, δ'-CH)/38.78; 4.13 (dd, α-CH)/63.93	✓	✓
44	Pyroglutamate	2.00 (m, β-CH_2); 2.40 (m, γ-CH_2)/32.27; 2.51 (m, β'-CH 2); 4.17 (dd, α-CH)	✓	✓
45	Pyruvate	2.36 (s, β-CH_3)	✓	✓

Table S1. (cont.)

No.	Compound	δ ^{1}H in ppm (multiplicity/ assignment)/ δ ^{13}C in ppm	Cell type	
			MDA-MB-231	MCF-10A
46	Serine	3.84 (dd, α-CH); 3.97 (m, β-CH_2)	✓	✓
47	Sorbitol	3.62 (m, C1H/C4H/C6H)/54.67, 3.73(d, C6H), 3.77 (m, C3H), 3.81 (d, C1H), 3.83 (m, C2H/C5H)	✓	✓
48	Succinate	2.41 (s, CH_2)	✓	✓
49	Taurine	3.27 (t, S-CH_2)/50.10; 3.42 (t, N-CH_2)/38.05	✓	✓
50	Threonine	1.32 (d, γ-CH_3)/22.62; 3.60 (d, α-CH)/62.97; 4.23 (m, β-CH)/67.27	✓	✓
51	Tryptophan	3.28 (dd, β-CH); 3.48 (dd, β'-CH); 4.01 (dd, α-CH); 7.21 (t, C5H, ring); 7.29 (t, C6H, ring); 7.32 (s, C2H, ring); 7.55 (d, C7H, ring); 7.74 (d, C4H,ring)	✓	✓
52	Tyrosine	3.06 (m, β'-CH); 3.19 (m, β-CH); 3.94 (m, α-CH); 6.90 (d, C3H, C5H, ring); 7.20 (d, C2H, C6H, ring)	✓	✓
53	UDP	4.26 (m, C5'H, ribose); 4.30 (m, C4'H, ribose); 4.38 (t, C2'H, ribose); 5.95 (s, C1'H, ribose); 5.97 (d, C6, ring); 8.00 (d, C5, ring)	✓	✓
54	UDP-N-acetyl-galactosamine (UDP-GalNAc)	5.56 (dd, C1'')	✓	✓
55	UDP-Glucose (UDP-Glc)	5.60 (dd, C1'');	✓	✓
56	UDP-N-acetyl-glucosamine (UDP-GlcNAc)	5.52 (dd, C1'')	✓	✓
58	UMP	3.99 (m, C5'H, ribose); 4.24 (m, C4'H, ribose); 4.41 (t, C2'H, ribose); 5.98 (m, C1'H, ribose/C6, ring); 8.12 (d, C5, ring)	✓	✓
59	UTP	4.25 (m, C4'H, ribose); 4.26 (m, C5'H, ribose); 4.43 (t, C2'H, ribose); 5.95 (s, C1'H, ribose); 5.97 (d, C6, ring); 7.98 (d, C5, ring)	✓	✓
60	Valine	0.99 (d, γ-CH_3)/19.21; 1.03 (d, γ'-CH_3)/20.48; 2.26 (m, β-CH); 3.59 (d, α-CH)/62.98	✓	✓

Table S2. Main metabolite variations in polar extracts of MDA-MB-231 cells exposed to 5 µM and 15 µM of BA and 10 µM and 20 µM of UA, in relation to controls, expressed as % variation (%var) and respective error (±), effect size (ES) and *p*-value (*p*). The variations with |ES| < 0.8 (or standard error > |ES|, or mean error > |% variation|) were considered null.

		BA				UA			
		5µM		15µM		10µM		20µM	
		48h	48h+24h	48h	48h+24h	48h	48h+24h	48h	48h+24h
Acetate	%var	0	0	21.07	22.70	0	0	10.69	11.02
	±			6.65	4.17			7.27	1.74
	ES	0	0	1.76	3.01	0	0	0.86	3.70
	p			0.0479	0.0145			0.2390	0.0033
Lactate	%var	53.08	0	55.73	0	0	0	0	0
	±	8.85		9.41					
	ES	2.91	0	2.85	0	0	0	0	0
	p	0.0115		0.0072					
Citrate	%var	-40.97	-12.45	-51.74	-19.78	-23.26	-10.99	-19.44	-15.02
	±	6.03	2.83	4.92	4.29	3.54	6.13	3.20	4.21
	ES	-5.25	-2.88	-8.72	-3.15	-4.58	-1.17	-4.14	-2.37
	p	0.0002	0.0169	1.0804E-05	0.0022	0.0014	0.1232	0.0052	0.0085
Fumarate	%var	-35.42	0	-37.50	0	0	0	-10.42	-10.34
	±	9.58		17.01				9.75	6.30
	ES	-2.76	0	-1.67	0	0	0	-0.69	-1.06
	p	0.0043		0.0497				0.3040	0.1817
Glu	%var	-24.61	0	-37.41	-11.90	0	0	0	0
	±	5.09		5.86	3.07				
	ES	-3.39	0	-4.83	-2.53	0	0	0	0
	p	0.0029		0.0003	0.0186				
Gln	%var	-28.49	0	-20.94	-5.26	-13.00	0	0	0
	±	5.90		6.99	4.20	7.36			
	ES	-3.46	0	-2.06	-0.79	-1.16	0	0	0
	p	0.0094		0.0172	0.2604	0.1079			
Asp	%var	-30.52	0	-12.45	0	0	0.00	24.90	32.91
	±	8.61		8.21				6.51	9.58
	ES	-2.57	0	-0.99	0	0	0.00	2.09	1.81
	p	0.0096		0.1632				0.0205	0.0390

Table S2. (cont.)

		BA				UA			
		5μM		15μM		10μM		20μM	
		48h	48h+24h	48h	48h+24h	48h	48h+24h	48h	48h+24h
Ala	%var	-12.64	-7.44	-10.34	-12.62	0	0.00	0	-12.11
	±	4.36	1.59	4.24	1.60				3.37
	ES	-1.90	-2.98	-1.58	-5.19	0	0.00	0	-2.35
	p	0.0219	0.0063	0.0429	0.0009				0.0162
Gly	%var	0	-8.57	-9.59	-31.75	0	-7.50	-15.08	-25.85
	±		1.94	6.90	1.84		2.44	5.06	1.37
	ES	0	-2.83	-0.90	-12.58	0	-1.96	-1.98	-13.32
	p		0.0052	0.1990	1.0429E-06		0.0297	0.0219	4.8081E-06
Pro	%var	16.73	0	21.93	0	0	0	0	0
	±	3.54		4.78					
	ES	2.68	0	2.54	0	0	0	0	0
	p	0.0112		0.0184					
Leu	%var	15.91	0	33.86	0	0	0	0	0
	±	2.07		6.04					
	ES	4.38	0	2.95	0	0	0	0	0
	p	0.0037		0.0112					
Ile	%var	14.68	0	30.97	0	0	0	0	0
	±	2.06		6.19					
	ES	4.08	0	2.66	0	0	0	0	0
	p	0.0044		0.0157					
Val	%var	18.46	0	38.79	0	0	0	0	0
	±	1.97		6.70					
	ES	5.28	0	2.98	0	0	0	0	0
	p	0.0008		0.0127					
Phe	%var	6.80	0	24.06	0	0	0	0	0
	±	2.51		6.82					
	ES	1.61	0	1.94	0	0	0	0	0
	p	0.0499		0.0403					

Table S2. (cont.)

		BA				UA			
		5μM		15μM		10μM		20μM	
		48h	48h+24h	48h	48h+24h	48h	48h+24h	48h	48h+24h
Tyr	%var	8.11	0	28.53	0	0	0	0	0
	±	2.49		6.45					
	ES	1.93	0	2.38	0	0	0	0	0
	p	0.0418		0.0202					
PCr	%var	-19.36	0	-22.44	-14.19	-13.59	0	-10.77	0
	±	4.46		5.44	4.07	4.18		5.40	
	ES	-2.95	0	-2.86	-2.31	-2.15	0	-1.30	0
	p	0.0077		0.0037	0.0134	0.0279		0.0797	
Cr	%var	22.45	0	18.99	0	0	0	0	0
	±	6.47		4.87					
	ES	1.92	0	2.19	0	0	0	0	0
	p	0.0274		0.0120					
ATP	%var	0	-7.54	0	-15.36	0	0	0	-9.33
	±		3.18		2.60				1.38
	ES	0	-1.52	0	-3.93	0	0	0	-4.37
	p		0.0814		0.0045				0.0007
Choline	%var	10.16	0	12.46	0	-6.23	0	0	0
	±	4.87		7.21		4.52			
	ES	1.22	0	1.00	0	-0.87	0	0	0
	p	0.0962		0.1752		0.2050			
PC	%var	-24.96	0	-38.62	0	-7.00	0	-6.17	21.66
	±	3.54		7.32		4.26		4.36	2.40
	ES	-4.96	0	-4.02	0	-1.05	0	-0.90	5.01
	p	0.0002		0.0029		0.1479		0.2042	0.0011
GPC	%var	13.50	0	26.27	0	9.94	0	10.72	-6.52
	±	2.21		5.30		3.24		5.81	2.25
	ES	3.52	0	2.69	0	1.80	0	1.08	-1.84
	p	0.0048		0.0125		0.0275		0.1575	0.0275

Table S2. (cont.)

		BA				UA			
		5µM		15µM		10µM		20µM	
		48h	48h+24h	48h	48h+24h	48h	48h+24h	48h	48h+24h
GSH	%var<br>±<br>ES<br>*p*	-15.77<br>4.65<br>-2.26<br>0.0223	0<br><br>0 	-25.87<br>6.40<br>-2.86<br>0.0035	0<br><br>0 	-7.40<br>5.98<br>-0.79<br>0.2457	0<br><br>0 	-7.50<br>5.29<br>-0.91<br>0.1939	-8.79<br>3.95<br>-1.43<br>0.0870
Tau	%var<br>±<br>ES<br>*p*	6.09<br>1.92<br>1.90<br>0.0407	0<br><br>0 	7.79<br>2.61<br>1.76<br>0.0561	0<br><br>0 	0<br><br>0 	0<br><br>0 	11.82<br>1.46<br>4.70<br>0.0013	0<br><br>0
myo-Ino	%var<br>±<br>ES<br>*p*	-8.23<br>1.20<br>-4.41<br>0.0011	-6.52<br>1.59<br>-2.61<br>0.0062	-18.58<br>2.70<br>-4.67<br>0.0014	-17.84<br>2.57<br>-4.69<br>0.0014	0<br><br>0 	0<br><br>0 	0<br><br>0 	-9.02<br>1.41<br>-4.13<br>0.0005

Table S3. Main metabolite variations in polar extracts of MCF-10A cells exposed to 5 µM and 15 µM of BA and 10 µM and 20 µM of UA, in relation to controls, expressed as % variation (%var) and respective error (±), effect size (ES) and *p*-value (*p*). The variations with |ES| < 0.8 (or standard error > |ES|, or mean error > |% variation|) were considered null.

		BA		UA			
		5µM		10µM		20µM	
		48h	48h+24h	48h	48h+24h	48h	48h+24h
Glucose	*%var*	-56.45	0	-71.26	-31.96	-85.89	-67.35
	±	29.74		15.82	16.86	15.82	21.76
	ES	-1.63	0	-4.30	-1.39	-5.85	-2.87
	p	0.0567		0.0007	0.0810	0.0007	0.0067
Glucose-1-phosphate	*%var*	-93.58	-92.25	-75.94	0	-78.61	-40.85
	±	16.11	35.07	17.96		12.98	16.06
	ES	-6.71	-3.00	-4.19	0	-6.14	-1.97
	p	0.0001	0.0057	0.0005		0.0006	0.0187
UDP-GlcNAc	*%var*	151.53	0	60.69	0	61.45	0
	±	9.25		10.15		6.32	
	ES	5.73	0	2.82	0	4.57	0
	p	0.0015		0.0128		0.0009	
UDP-GalNAc	*%var*	123.58	0	62.26	0	68.87	0
	±	13.12		12.79		12.12	
	ES	3.58	0	2.28	0	2.60	0
	p	0.0026		0.0109		0.0060	
UDP-Glucose	*%var*	-75.09	-66.89	-19.78	0	-32.23	-20.07
	±	9.60	20.53	6.86		7.47	4.37
	ES	-7.70	-3.01	-1.97	0	-3.16	-3.14
	p	0.0000	0.0139	0.0187		0.0021	0.0024
Lactate	*%var*	161.49	0	0	0	0	0
	±	34.30					
	ES	1.60	0	0	0	0	0
	p	0.0786					
Citrate	*%var*	-74.68	-61.27	-36.91	-18.09	-38.52	-32.19
	±	3.76	5.96	3.57	8.81	3.85	8.84
	ES	-19.48	-9.11	-7.80	-1.39	-7.61	-2.67
	p	2.8047E-06	0.0001	0.00002	0.0943	0.00002	0.0144

Table S3. (cont.)

		BA			UA		
		5μM		10μM		20μM	
		48h	48h+24h	48h	48h+24h	48h	48h+24h
Fumarate	*%var*	-80.00	0	0	-22.45	0	-34.69
	±	54.43			17.15		14.18
	ES	-1.51	0	0	-0.91	0	-1.82
	p	0.0849			0.1908		0.0401
Formate	*%var*	91.07	287.80	0	0	0	0
	±	13.94	19.51				
	ES	2.76	3.72	0	0	0	0
	p	0.0092	0.0086				
3-Hydroxyisobutyrate	*%var*	-48.45	-48.51	-23.71	-22.77	-30.93	-30.69
	±	8.28	6.18	8.07	7.27	7.11	6.58
	ES	-4.75	-6.38	-2.05	-2.17	-3.16	-3.39
	p	0.0012	0.0006	0.0176	0.0124	0.0082	0.0018
Acetate	*%var*	33.50	63.78	0	0	0	10.61
	±	7.76	13.91				3.19
	ES	2.27	2.14	0	0	0	1.94
	p	0.0138	0.0389				0.0236
Glu	*%var*	-75.91	-47.27	-21.17	0	-23.01	-11.32
	±	8.73	8.30	6.62		6.62	7.41
	ES	-8.61	-4.59	-2.20	0	-2.41	-1.00
	p	0.00003	0.0005	0.0128		0.0088	0.1665
Gln	*%var*	0	0	0	16.14	0	25.50
	±				2.45		2.43
	ES	0	0	0	3.75	0	5.72
	p				0.0023		0.0004
Asp	*%var*	-48.70	-24.38	-38.30	-26.58	-46.10	-37.26
	±	4.01	14.06	4.21	9.00	4.90	10.00
	ES	-9.87	-1.21	-6.92	-2.09	-7.52	-2.82
	p	0.0003	0.1111	0.0001	0.0146	0.00003	0.0038

Table S3. (cont.)

		BA			UA		
		5µM		10µM		20µM	
		48h	48h+24h	48h	48h+24h	48h	48h+24h
Ala	*%var*	-39.74	-65.18	44.32	0	0	0
	±	10.37	7.36	11.33			
	ES	-2.94	-8.08	1.97	0	0	0
	p	0.0031	0.0001	0.0299			
Gly	*%var*	41.70	0	40.31	24.70	38.29	31.50
	±	5.34		4.91	6.74	4.53	5.56
	ES	3.97	0	4.20	2.01	4.36	3.01
	p	0.0010		0.0006	0.0435	0.0004	0.0137
Pro	*%var*	80.04	0	87.39	49.66	69.18	78.81
	±	5.11		3.36	7.94	5.64	6.79
	ES	6.88	0	11.13	3.08	5.60	5.12
	p	0.0003		4.9446E-06	0.0102	0.0009	0.0018
His	*%var*	-25.16	-44.80	0	0	0	0
	±	9.10	4.76				
	ES	-1.95	-7.45	0	0	0	0
	p	0.0281	0.0001				
Leu	*%var*	418.85	75.04	389.84	82.57	297.70	130.47
	±	9.04	8.46	10.01	9.08	15.33	6.93
	ES	9.21	3.97	8.12	3.96	4.80	7.01
	p	0.0002	0.0020	0.0001	0.0010	0.0002	0.0002
Ile	*%var*	218.16	36.26	211.80	58.71	156.93	94.71
	±	6.74	8.03	8.75	8.29	13.07	6.37
	ES	9.52	2.35	7.23	3.37	4.14	6.20
	p	0.0003	0.0149	0.00003	0.0018	0.0008	0.0003
Val	*%var*	138.35	10.36	151.36	47.98	101.22	70.45
	±	7.05	7.17	7.07	7.20	10.19	6.25
	ES	7.14	0.84	7.50	3.31	4.05	5.12
	p	0.0005	0.2444	0.0002	0.0021	0.0006	0.0003

Table S3. (cont.)

		BA			UA		
		5µM		10µM		20µM	
		48h	48h+24h	48h	48h+24h	48h	48h+24h
Phe	*%var*	0	-24.03	22.52	16.59	0	27.29
	±		3.87	4.02	5.94		5.68
	ES	0	-4.33	3.10	1.59	0	2.60
	p		0.0007	0.0129	0.0563		0.0112
Tyr	*%var*	-16.91	-36.13	0	13.28	0	21.23
	±	6.33	4.38		6.11		6.07
	ES	-1.79	-6.19	0	1.25	0	1.95
	p	0.0306	0.0002		0.1023		0.0290
PCr	*%var*	0	0	34.32	24.82	0	0
	±			5.07	8.78		
	ES	0	0	3.55	1.55	0	0
	p			0.0066	0.0625		
Cr	*%var*	59.47	0	-16.76	0	0	28.29
	±	7.16		7.24			5.90
	ES	3.94	0	-1.55	0	0	2.32
	p	0.0010		0.0504			0.0233
ATP	*%var*	0	0	41.52	0	53.13	0
	±			8.28		4.46	
	ES	0	0	2.55	0	5.79	0
	p			0.0099		0.0003	
NADH	*%var*	62.88	0	34.09	20.35	46.21	55.81
	±	9.38		7.12	12.26	10.93	12.96
	ES	3.14	0	2.52	0.93	2.11	2.07
	p	0.0030		0.0095	0.1871	0.0185	0.0155
1-Methylnicotinamide	*%var*	152.75	56.61	38.46	22.91	83.24	50.44
	±	6.98	12.39	4.12	11.32	5.37	11.40
	ES	7.62	2.19	4.81	1.12	6.73	2.17
	p	0.0006	0.0194	0.0003	0.1249	0.0003	0.0174

Table S3. (cont.)

		BA			UA		
		5μM		10μM		20μM	
		48h	48h+24h	48h	48h+24h	48h	48h+24h
Choline	*%var*	46.22	0	31.99	0	30.58	0
	±	5.48		10.12		5.34	
	ES	4.21	0	1.68	0	3.05	0
	p	0.0005		0.0515		0.0027	
PC	*%var*	-78.42	0	-26.30	-38.35	-17.27	-38.16
	±	9.97		10.76	11.54	8.07	8.48
	ES	-7.96	0	-1.73	-2.53	-1.44	-3.42
	p	0.00004		0.0360	0.0081	0.0578	0.0016
GPC	*%var*	307.70	188.81	108.51	-32.45	160.13	0
	±	7.34	17.03	7.69	11.53	8.43	
	ES	10.15	3.51	5.63	-2.07	6.49	0
	p	0.0003	0.0068	0.0015	0.0364	0.0012	
GSH	*%var*	-31.85	-24.73	-30.60	-27.29	-21.64	-26.66
	±	4.66	20.84	5.71	11.44	4.28	12.31
	ES	-4.99	-0.83	-3.89	-1.70	-3.48	-1.54
	p	0.0003	0.2549	0.0008	0.0382	0.0019	0.0559
myo-Lno	*%var*	-19.23	-52.77	-13.14	0	0	0
	±	5.02	9.47	3.34			
	ES	-2.60	-4.65	-2.59	0	0	0
	p	0.0090	0.0022	0.0057			
Dimethylamine	*%var*	-40.51	0	-53.26	-15.34	-41.93	-23.93
	±	17.23		17.87	8.94	17.00	8.69
	ES	-1.81	0	-2.50	-1.14	-1.92	-1.92
	p	0.0480		0.0266	0.1253	0.0458	0.0379

Table S4. Assignment of resonances in the NMR profile of organic extracts from MDA-MB-231 cells and MCF-10A cells. Multiplicity: s, singlet; d, doublet; t, triplet; m, multiplet; dd, double of doublets.

No.	Compound	δ ^{1}H in ppm (multiplicity/ assignment)/ δ ^{13}C in ppm	Cell type	
			MDA-MB-231	MCF-10A
1	Cholesterol	0.69 (s, CH_{3}-18)/11.84; 0.85 (d, CH_{3}-26)/22.54; 0.89 (d, CH_{3}-27)/22.54; 0.89(d, CH_{3}-21)/18.54; 0.92 (m, CH-9)/50.02; 0.98 (m, CH-14)/56.92; 0.99 (s, CH_{3}-19)/19.21; 1.06 (s, CH-15)/24.26; 1.06 (m, CH_{2}-1)/37.15; 1.07 (m, CH-17)/56.07; 1.11 (m, CH_{2}-24)/39.45; 1.12 (m, CH_{2}-23)/23.71; 1.32 (m, CH-20)/35.90; 1.47 (m, CH_{2}-11)/20.94; 1.49 (m, CH_{2}-2)/31.67; 1.50 (m, CH-25)/27.82; 1.94 (t, CH_{2}-7)/31.94; 1.97 (t, CH_{2}-7')/31.94; 1.98 (CH_{2}-12)/39.63; 2.00 (CH_{2}-12)/39.58; 2.26 (m, CH_{2}-4')/42.17; 3.51 (m, CH-3)/71.68; 5.33 (m, CH-6)/121.65	✓	✓
2	Cholesterol ester	1.02 (s, CH_{3}-19); 1.83 (m, CH_{2}-2); 1.84 (m, CH_{2}-1)/37.10; 2.31 (m, CH_{2}-4'); 4.61 (m, CH-3)	✓	✓
3	Diglycerides	3.73, 4.28 (glyceryl CH_2 *sn*1/*sn*3); 5.08 (glyceryl CH *sn*2)	✓	✓
4	Fatty acyl chains (mainly in phospholipids)	0.87 (t, $CH_3(CH_2)_n$)/13.98; 1.27 (m, $(CH_2)_n$)/22.54/29.55/31.71; 1.57 (m, $-CH_2-CH_2CO$)/24.80; 2.03 (m, $-CH_2CH=$)/27.07; 2.28 (m, $-CH_2COOR$)/34.06; 2.80 (t, $=CHCH_2CH=$)/25.50; 5.35 (m, -HC=CH-)/129.03	✓	✓
5	Free fatty acids	0.98 (t, $CH_3 (CH_2)_n$); 1.61 (m, $-CH_2-CH_2CO$); 2.05 (m, $-CH_2CH=$); 2.35 (t, $-CH_2COOH$); 2.76 (t, $=CHCH_2CH=$)	✓	✓
6	Phosphatidylcholine (PTC)	3.30 (s, $N(CH_3)_3$)/54.44; 3.75 (CH_2N)/66.45; 3.92 (glyceryl CH_2 *sn*3)/63.70; 4.38 (glyceryl CH_2 *sn*1)/62.77; 4.31 (CH_2-OP)/59.33; 5.19 (glyceryl CH *sn*2)/70.27	✓	✓
7	Phosphatidylethanol-amine (PTE, diacyl form)	3.15 (s, CH_2 -N)/40.46; 3.55 (glyceryl CH_2 *sn*1)/69.05; 3.92 (glyceryl CH_2 sn3)/63.58; 4.11 (CH_2 -OP)/62.66; 5.19 (glyceryl CH sn2)/70.24	✓	✓
8	PTE plasmalogen (PTE, plasmenyl form)	1.27 ($(CH_2)_n$); 2.00 ($-CH=CH-CH_2$); 3.90 (glyceryl CH_2 *sn*3); 4.32 (glyceryl CH_2 *sn*1); 5.90 (-CH=CH-)	✓	✓
9	Sphingomyelin (SM)	3.33 (s, $N(CH_3)_3$); 5.45 (-CH=CH-); 5.67 (-CH=CH-)	✓	✓
10	Triglycerides (TG)	4.13, 4.27 (dd, glyceryl CH_2 *sn*1/*sn*3)/61.93; 5.19 (glyceryl CH *sn*2)/70.26	✓	✓
